# Trade openness and economic growth in Africa's regional economic communities: empirical evidence from ECOWAS and SADC

**DOI:** 10.1016/j.heliyon.2021.e06996

**Published:** 2021-05-14

**Authors:** Betsy M. Oloyede, Evans S. Osabuohien, Jeremiah O. Ejemeyovwi

**Affiliations:** a Department of Economics & Development Studies, Covenant University, Ota, Nigeria; b Centre for Economic Policy and Development Research-CEPDeR, Covenant University, Ota, Nigeria; c Department of Economics & Development Studies & Research Fellow at CEPDeR, Covenant University, Ota, Nigeria; d Statistics Department, Central Bank of Nigeria, Lagos, Nigeria

**Keywords:** ECOWAS, FDI, Regional economic community, SADC, Trade openness

## Abstract

In light of increasing globalisation, countries do not just open their economies to trade; some factors have to be influenced. This study analyses the relationship among trade openness and macroeconomic outlook of Africa's regional economic communities (RECs), focusing on the Economic Community of West African States (ECOWAS) and Southern African Development Community (SADC). The study applies the Pooled OLS, Fixed and Random Effects techniques of estimation and the Durbin-Wu Hausman test for endogeneity to categorised secondary data from the World Bank's World Development Indicators (WDI) and the United Nations Conference on Trade and Development (UNCTAD) databases. The datasets are classified into three segments for comparative analysis, comprising the total, ECOWAS, and SADC datasets. The results show a positive but insignificant nexus between economic growth rate and trade openness in both the combined simulated ECOWAS and SADC and the individual REC. The results emphasise that the government and other relevant stakeholders should ensure policies are enacted and enforced to transmit the experienced economic growth into substantial trade gains and further trade openness in ECOWAS and SADC.

## 1 Introduction

The study examines the impact economic growth has on trade openness in Africa's regional economic communities (RECs) drawing empirical evidence from Economic Community of West African States (ECOWAS) and Southern African Development Community (SADC). This objective is motivated by the extant literature on the role of trade openness on economic growth and the argument on the essence of economic growth on trade openness. The study questions if economic growth in the selected RECs significantly transmits to impact the trade openness. The ECOWAS and SADC are chosen as the study's focus because, among the existing RECs in Africa, ECOWAS and SADC are two of the most successful regional integration agreements with large imports and exports of both financial and non-financial goods and services (Ejemeyovwi et al., 2018, World Bank, 2019). Hence, these selected RECs provide good samples to investigate the impact of economic growth on trade openness, replicable to the other Africa's RECs.

Owing to the increasing realisation of facts by leaders in Africa, the continent's sectoral fragmentation's value is rising, and the disassociation from global markets is in many ways turning into something reasonable. Some countries relish a large amount of profit from traditionally high oil costs, whereas several others struggle with the challenges and shortage of power supply. Some also suffered from production deficiency because of high prices and decreased dependability on the transportation system as well as weak infrastructure, which typically limits intra-African trade (World Bank, 2019). Mengesha (2009) opines that this additionally attracts the attention to the very fact that Africa is faced with impoverishment, a small percentage of global trade, and slow progress in the development of infrastructure. In an effort for economies to increase economic progress and drive international trade, regional trade agreements (RTAs) in the previous decades, have been increasingly embraced by several economies in Africa. These regional agreements possess the ambition of facilitating trade among countries by reducing costs of transaction like tariff and non-tariff barriers and breaking the limitation of cooperation among countries towards achieving the aim of an African Common Market (Foroutan, 1992; Olayiwola et al., 2015; Osabuohien et al., 2019).

Regional integration and by implication, trade liberalisation is not without consequences and impact. Some critics posit that the introduction of free trade leads to a hike in social and environmental challenges, particularly in developing countries. Furthermore, regardless of the perceived benefits of the north-south cooperation, incomes of most of the sub-Saharan African (SSA) countries either dwindled or remained stagnant. A significant part of this low-level economic development was that these economies were reliant on high-cost western technology that were not suitable or did not match their primary production systems (Osabohien et al., 2021).

To promote regional integration and trade, the African Union has put together the African Continental Free Trade Area (AfCFTA). The AfCFTA aims at: The Development of a single market in Africa; Engulf a market of 1.2 billion people with the sum of US$2.5 trillion as its gross domestic products (GDP); Cause intra-African trade to accelerate up to 52.3%; Allow all member countries of the AU share in the gains regarding welfare for the nations estimated at around 2.64% of African GDP - roughly $65 billion in 2018 terms; Bring about the improvement of real wages for unskilled workers in major sectors such as the agricultural and non-agricultural sectors, not neglecting skilled workers; Make room for additional dynamic benefits, such as, long-lasting and sustained growth, diversification of export, a vast African market that attracts foreign direct investment better, with a more spacious economic atmosphere for industrialisation and catalytic effects for structural transformation; and Enlarge the size of the African economy to twenty-nine trillion US dollars by 2050. Having stated the objectives of the AfCFTA, it is essential to examine how macroeconomic conditions influence trade openness in Africa's RECs drawing empirical evidence from ECOWAS and SADC countries.

Therefore, countries do not just open their economies for foreign trade; many influencing factors are to be considered. To this end, it is imperative to examine how the macroeconomic conditions influence trade openness in ECOWAS and SADC. A couple of studies have investigated trade openness and the selected macroeconomic variables in Africa. However, the bulk of the literature has been concerned about the reverse case. In achieving its objective, this study utilises the pooled ordinary least squares (OLS) technique, the fixed effect and random effect estimation techniques. The study further employs the Durbin-Wu Hausman test for endogeneity, Breusch Pagan Lagrange Multiplier test for random effects and the Hausman test to ensure that there is absence of endogeneity and decide the most estimation technique to report. The study finds that economic growth does not significantly affect trade openness in the combined RECs as well as ECOWAS and SADC. This study argues that an improvement in nations' economic conditions will improve the critical structure that will enable the countries to embrace trading more with each other and motivate them to be more open to trade.

## 2 Insights from the literature

There has been an increase in scholarly works on trade openness, macroeconomic variables and regional economic communities over the years. Some of the empirical results and their methodologies are discussed herewith. Osabuohien (2007) analysed how trade openness influences the economic performance of ECOWAS members. The study focused on Ghana and Nigeria using Augmented Dickey-Fuller and Philip Perron stationarity tests, co-integration and vector error correction techniques. In the findings, trade openness and real government expenditure were seen to be positively impactful on the selected ECOWAS countries' economies.

In addressing trade creation and diversion intensities, Musila (2005) analysed data for 20 African countries (1991–1998) using the gravity model to ascertain the trade creation and trade diversion intensities in COMESA, ECCAS and ECOWAS, and concluded that the trade creation and trade diversion differed by region and by period. For instance, the study found out that the intensity of trade creation was very high in ECOWAS followed by COMESA. ECCAS, on the other hand, did not show a stable indication of trade creation or trade diversion effect. Coulibaly (2008), also using panel data analysed the trade effects of 22 RTAs including SSA RTAs (1962–2006), adopted the gravity equation's aid in a Hausman-Taylor specification. The study discovered a positive impact on member countries intra-regional exports. (Olayiwola and Busari, 2008) also examined African Economic Growth's Trade Strategy and Trade Facilitation using the ECOWAS Scenario. The study showed that Nigeria's real Gross Domestic Products (GDP) did not substantially affect the import value index of Nigeria from Ghana. ECOWAS membership also had an insignificant adverse association with Nigeria's import value index from Ghana but had a substantial favourable relationship with Nigeria's exports to Ghana. Also, government expenditure on infrastructure, the gap in real gross GDP per capita between Ghana and Nigeria, the Ghanaian population, and the real GDP of the two countries are significant determinants of these countries' trade capacity. The study concluded that trade facilitation is needed to facilitate inter-trade and intra-trade between ECOWAS countries.

The effect of the North American Free Trade Agreement (NAFTA) on trade between its member states was also investigated by Gould (2009). The author used the theoretical basis of the gravity model by Bertrand (1985) which derives from the assumptions that producers optimise benefit subject to a constant elasticity of substitution efficiency and a budget limit. For leverage over market increases and multilateral opposition, exchange rates were included. Gould (2009) found evidence that NAFTA had a significant positive effect on trade flows between the US and Mexico, using pre and post-NAFTA quarterly trade data (1986–1996). Nevertheless, this was not the same for trade between the USA and Canada or Canada and Mexico because of already established bilateral trade relations. While the North-South RTAs have successfully increased trade between countries, developed countries have continued to rely on political and economic independence for themselves. South-South partnership theories became essential parts of most developed countries' economic strategies in the late 1970s. By building trading relations with other developed countries rather than dependent on the wealthy North, most developing countries found that they were more likely to benefit from low-cost solutions to their economic development problems. Consequently, regional integration became a key strategy which allowed first Asia, and then other developing regions, to turn their small and mostly agricultural economies into more highly industrialised economies by benefiting from economies of scale.

Eris and Ulasan (2013) examined the interaction between trade openness and long-run economic growth using the Bayesian model averaging techniques to systematically account for uncertainty in the model. The study found a result that showed robust inclusion of varying proxies of trade openness and none on the proxies were connected with economic growth robustly. Semancikova (2016) examined the link among trade, trade openness and macroeconomic performance using descriptive and comparative analysis of significant empirical studies. The empirical results indicate a positive effect of trade and trade openness on macroeconomic variables. At the same time, Bagnai et al. (2016), used the post-Keynesian balance-of-payments constrained growth approach while focusing on South-South trade's contribution to examine the then increasing growth in the economy of Sub-Saharan Africa (SSA). The study adopted panel co-integration method of estimation to carry out the analysis which focused on 20 low-income and lower-middle-income SSA countries, using annual data from 1990 to 2008. The results revealed that there had been a relaxation in constraints of balance-of-payments of SSA.

Studies such as Yang and Gupta (2008) concluded that trade agreements within SSA have not been trade-enhancing among member countries. Contrary to these sceptics of African RTAs, studies by Carrère (2004), Musila (2005), Coulibaly (2008), Afesorgbor and Van Bergeijk (2011) and Turkson (2012) have shown that African RTAs positively impact on bilateral trade flows except a few RTAs in their analysis which have had compliance problems with member countries. For instance, Afesorgbor and Van Bergeijk (2011) showed that ECOWAS and SADC had a positive intra-regional trade compared to the arrangement with the EU and concluded that the enhancing trade effect of the regional integration analysed was more potent than that of the EU. Similarly, Turkson (2012) found a positive trade effect within ECOWAS, EAC, and SADC compared to trade agreements between SSA and the EU but a negative trade effect in ECCAS and IGAD.

Alege and Osabuohien (2015) explored the interaction between exchange rate and international rate trade in SSA. The authors developed, based on two equations and in these equations exchange rate, real gross domestic product (GDP), stock of capital and technology were the explanatory variables. The analysis carried out showed that exports and imports are unresponsive to changes in the exchange rate. It followed that considering the view of the economic structures and compositions of export, depreciation of currencies in the region might not have the expected results. Similarly, the balance of payments may be worsened, but depreciation would not depress imports.

From the reviewed studies in the investigation of trade openness and the selected macroeconomic variables in Africa, the comparative study of ECOWAS and SADC has not been sufficiently explored to the best of the researchers’ knowledge, and the variable-trade openness would be treated as the dependent variable as against the literature trend. Therefore, this study investigates the issues to understand the influence of current comparative dynamics on the subject.

## 3 Methodology

### 3.1 Empirical model

The model specification is adapted from the model of Osabuohien and Efobi (2014), which specified a model that expressed trade openness as a function of economic performance, exchange rate, official lending rate, foreign reserves and foreign direct investment. This study improves Osabuohien and Efobi (2014)'s model by first, introducing dummy variables to deal with the impact of the regional economic blocks and second, focusing on the comparative analysis of SADC and ECOWAS, which are two leading regional economic blocks in Africa with impressive trade performance and economic growth; and third, comparing the result of the individual RECs to the findings of the combined REC (capturing if ECOWAS and SADC were to merge under any circumstance). This is necessary as most African countries are involved in regional economic integration to improve trade performance. Also, most African countries have experienced growth overtime, but whether this experienced economic growth also transmits into trade openness, exists in policymakers and researchers.

Following Osabuohien and Efobi (2014), trade openness in this study is measured by the ratio of trade volume to the gross domestic product (GDP). This is a valid measure as it reflects a country's actual degree of integration into the world economy (Grabner et al., 2020). Grabner et al. (2020) also categorised the measure as ‘de-facto’ given that focus on the economic and statistical indication of trade openness. However, the authors acknowledge that the weakness of this ‘trade openness’ measure is that the ratio of trade volume to GDP does not capture the legal framework behind the trade volume, which can be captured by tariff rates and non-tariff trade barriers, which is captured in the ‘de-jure’ measures. This study for robustness would have utilised the ‘de-jure’ measure also, but due to data paucity for the selected regions of interest, the study focuses on the ‘de-facto’ measures.

Notably, economic growth is introduced in this study as an explanatory variable that captures the performance of most macroeconomic variables, which include labour, population and employment. These variables are subsumed in the economic growth measure to avoid possible endogeneity and multicollinearity issues. The study further included only the necessary explanatory variables relevant to determining trade openness to the best of the authors' knowledge. This study's other explanatory variables include the exchange rate, foreign reserve, foreign direct investment (FDI) net inflows, lending rate, and dummy variables for RECs identification.

The empirical model of this study is therefore specified implicitly as;

(1) opn=f(grt,exc,frs,fdi,lendr,recdum,ε)

Eq. (1) is re-written explicitly as

(2) $$opn_{it}=\;grt_{it}^{\beta_{1}}exc_{it}^{\beta_{2}}frs_{it}^{\beta_{3}}fdi_{it}^{\beta_{4}}lendr_{it}^{\beta_{5}}\;exp^{\beta_{6}recdum}\varepsilon_{it}$$

The study takes the natural logarithm of the empirical model to avoid econometric concerns, which include the non-linear distribution of the data, catering for possible heteroskedasticity, to smoothen the data and to ensure that the derived policy estimates are interpreted in the form of elasticities – the rate of change in one variable as a result of the introduction of a unit or percentage change in an explanatory variable”. Also notable was that the authors applied the natural logarithm to the absolute values FDI net inflow due to the authors' inability to successfully log the negative figures. This action is justifiable as the negative FDI figures indicate that the outflows are more in quantity than the inflows into a country. This is a common characteristic among many African countries.

The explicit model after taking the natural logarithm is expressed as:

(3) $$lnopn_{it}=\;\beta_{0i}+\beta_{1}lngrt_{it}+\;\beta_{2}lnexc_{it}+\;\beta_{3}lnfrs_{it}+\;\beta_{4}lnfdi_{it}\;+\;\beta_{5}lnlendr_{it}+\;\beta_{6}recdum+\varepsilon_{it}$$

where: *lnopn*_*it*_; natural logarithm of Openness of country *i* at period *t*; *lngrt*_*it*_; natural logarithm of the economic growth rate of country *i* at period *t*; *lnexc*_*it*_; natural logarithm of the official exchange rate of country *i* at period *t*; *lnfrs*_*it*_; natural logarithm of foreign reserve of country *i* at period *t*; *lnfdi*_*it*_; natural logarithm foreign direct investment of country *i* at period *t; lnlendr*_*it*_; natural logarithm of lending rate of country *i* at period *t; recdum;* dummy variables for the Regional Economic Communities (RECs), where ECOWAS is represented as 1 and SADC 0, which cannot accommodate logarithm. Hence the introduction of the exponential function. β_0i_; intercept of the model; β_1 – 5_: represents the coefficients of the independent variables; *it*: individual country and time identifier (*i* = 15, *t =* 12).

### 3.2 Technique of estimation

#### 3.2.1 Pooled ordinary least squares

A pooled model is used under the assumption that the individuals behave in the same way, where there are homoscedasticity and no autocorrelation. Only then OLS can be used for obtaining efficient estimates. The pooled model's assumptions are the same as for the simple regression model, which include the absence of multicollinearity, endogeneity, heteroscedasticity, cross-section or time-series correlation, and non-normal distribution of the disturbances ϵ_*it*_ (Greene 2012).

A pooled model can be expressed as:

(4) $$lnopn_{it}=\;\beta_{0i}+\beta_{1}lngrt_{it}+\;\beta_{2}lnexc_{it}+\;\beta_{3}lnfrs_{it}+\;\beta_{4}lnfdi_{it}\;+\;\beta_{5}lnlendr_{it}+\;\beta_{6}recdum+\;\varepsilon_{i}$$

These assumptions of the common OLS are also determinative for the panel data models. In studies involving panel data, there is a possibility that there would be an occurrence of autocorrelation of the disturbances within individuals. There is, however, a possibility that would cause tendentious estimates of the standard errors. There will be the existence of underestimation resulting in t-statistics that are over-estimated. The error should be adjusted, and one way to do so is by the utilisation of clustered standard errors which is the major problem with this model. Nonetheless, that does not distinctively differentiate the variables based on their peculiarities. In other words, Pooled OLS lumps them together and ignores the heterogeneity that exists among the countries. This may result in the model's estimated coefficients being bias and inconsistent, as the case may be.

#### 3.2.2 Fixed effect model

Fixed Effects (FE) model helps in the analysis of the effect of variables that are time-invariant. FE explores the connection between independent and predicted variables within an entity. Fixed effects model permits consideration of the heterogeneousness that exists among each individual (in this case, each country), by making room for each entity to own its intercept value. The term 'fixed effects' is concerned with the fact that although the intercept could vary across countries, each individual's intercept does not change overtime. It also assumed that the slope coefficients of the regressors do not vary across countries or overtime.

The fixed effects (FE) model helps in the analysis of the impact of time-invariant variables. FE investigates the relation within an organism between independent and expected variables. The fixed effects model allows for the variability that occurs within each person (in this case, each country) to be taken into account by allowing space for each entity to own its intercept value. The word 'se-results' is associated with the fact that whilst the intercept may differ across countries, each individual's intercept does not change overtime. The regressors' slope coefficients were also believed not to differ across countries or overtime (Gujarati and Porter, 2009; Ejemeyovwi et al., 2019). A typical fixed effects model takes this form:

(5) $$lnopn_{it}=\;\beta_{0i}+\beta_{1}lngrt_{it}+\;\beta_{2}lnexc_{it}+\;\beta_{3}lnfrs_{it}+\;\beta_{4}lnfdi_{it}\;+\;\beta_{5}lnlendr_{it}+\;\beta_{6}recdum+\varepsilon_{it}$$

#### 3.2.3 Random effects model

The distinguishing factors across entities in a random effects model are assumed to be unrelated to the model's predictors and error term. One of the benefits of random effects is that it also includes time-invariant variables (i.e., gender). In the fixed effects model, these variables are absorbed by the intercept while in random effects model, elements that are individual-specific are not treated as a parameter and are not being estimated. Instead, it is considered a random variable with mean and variance (Ejemeyovwi et al., 2019). However, the random effects model is represented, thus;

(6) $$lnopn_{it}=\;\beta_{1}lngrt_{it}+\;\beta_{2}lnexc_{it}+\;\beta_{3}lnfrs_{it}+\;\beta_{4}lnfdi_{it}\;+\;\beta_{5}lnlendr_{it}+\;\beta_{6}recdum+\varepsilon_{it}+\;\alpha +\mu_{it}+\varepsilon_{it}$$

#### 3.2.4 Hausman test

The Hausman test is used to determine which of the Fixed Effects Technique (FE) or Random Effects Technique (RE) methods are sufficient for this analysis. The model's choice to be used in a panel analysis using the fixed or random effect model is expected to be based on information about the distinctive components and the exogeneity of the independent variables. Some hypotheses are tested, and the outcomes are used to select the appropriate model. One of them is for testing whether fixed or random effects model is appropriate, by identifying endogeneity in the explanatory variables: Hausman test. The random effects model, when used appropriately, exhibits the best linear unbiased estimates (BLUE) properties (Sheytanova, 2015). Nevertheless, if the existence of correlation is found between the stochastic term of the random effects model and the independent variables, there would be inconsistency in its estimates, resulting in a preference for the fixed effects model (Ejemeyovwi et al., 2019). At times, in the random effects model, the dynamic component α might be related to the independent variable. The fixed effects model estimates are invariably consistent, yet not wholly efficient than the random effects model estimates. These properties of the panel data model estimates made this study to adopt the Hausman test.

### 3.3 Variables and data measurement

This study covers 31 African countries, comprising of 15 ECOWAS and 16 SADC members for the period 2006 to 2017. The list of countries with respect to their respective membership of ECOWAS and SADC are in Table A1 inAppendix. The period utilised by this study was informed by the economic events Africa before the signing of the African Continental Free Trade Area (AfCTA) proposed in 2015 and signed in 2018; which include the significant improvement in Africa's trade as a result of technological globalisation in terms of the vast and general adoption of information and communication technology within the region (Ejemeyovwi et al., 2020; Ejemeyovwi and Osabuohien, 2020). These reasons alongside data paucity led to utilising the period to truly capture the effects of economic growth on trade openness in Africa.

The selection of ECOWAS and SADC was motivated by the fact that ECOWAS and SADC are two of the most vibrant regional economic communities (RECs) with huge imports as well as exports of financial and non-financial goods and services (World Bank, 2019). The selection was also motivated because ECOWAS and SADC account for 80% of Sub-Saharan Africa's total trade flows (International Trade Centre, 2017) . The utilised data was sourced from World Development Indicators (World Bank, 2019) and United Nations Conference Trade and Development statistics databases (UNCTAD). The variables used by the study include trade openness, economic growth rate, exchange rate, foreign reserve, foreign direct investment and lending rate. More details of the variables are presented in Table 1.

**Table 1:** Data sources and measurement.

Data	Variable Definition	Measurement	Data Source
Trade Openness (*opn*_*it*_)	Trade Openness is the total of exports and imports of goods and services measured as a share of GDP i.e., import plus export divided GDP. As the exchange rate depreciates, it is expected that the country's export will be higher, which means more trade in the economy. For the robustness tests, the world trade share and composite trade share are interchangeably utilised to measure trade openness.	Ratio	World Bank (2019)
Economic Growth rate (*grtr*_*it*_)	The growth of an economy is measured using the change in the volume of its output or the real earnings of its residents*.* Economic growth rate is the annual proportion of the value of GDP growth rate at market prices based on constant local currency	Percentage	World Bank (2019)
Real effective exchange rate (*exc*_*it*_)	Exchange rate is the amount per unit of one currency to another. Exchange rate represents the real rate of domestic currency to the United States Dollar	US Dollars	UNCTAD STAT (2018)
Foreign Reserve (*fruit*)	Foreign reserves include exclusive drawing rights, reserves retained by creditors of the International Monetary Fund (IMF) and foreign exchange portfolios under the monetary authorities' supervision. Holdings of gold are exempted.	US Dollars	World Bank (2019)
Foreign direct investment net inflow(*fdi*_*it*_)	Foreign direct investment refers to an investment made by a wholly or partially foreign-owned company. It is a foreign investment in which a citizen (direct investor) in one country acquires a permanent stake in a business in another country (the direct investment enterprise).	US Dollars	World Bank (2019)
Lending rate (*lendr*_*it*_)	The lending rate is the bank rate used to satisfy the private sector's short- and long-term monetary needs. This rate often varies according to how creditworthy the investors are and the lending goals. These prices have terms and conditions added to them, and they differ from country to country, thereby restricting their comparability.	Percentage	World Bank (2019)
Recdum	The dummy variable is a proxy for regional economic communities to control for the effect of RECs.	Binary digits	Authors' computation

## 4 Results and discussion

### 4.1 Descriptive analysis

The description of the actual variables utilised in the analysis is discussed herewith. The dependent and independent variables utilised include economic growth rate, trade openness, exchange rate, foreign reserve, foreign direct investment, and lending rate. Notably, trade openness was captured by trade to GDP ratio, world trade share and composite trade share. The natural logarithms of the variables were taken to avoid econometric problems in the estimation model. The descriptive information is displayed in Table 2.

**Table 2:** Descriptive statistics.

Variable	Observation	Mean	Min	Max
**ECOWAS and SADC COMBINED**				
Trade to GDP ratio	370	81.08	20.72	311.35
Economic Growth	371	4.27	-20.59	20.71
Exchange Rate	372	657.86	0.92	9100
Foreign Reserve	302	3410000000	0.02	45500000000
Foreign Direct Investment	372	841000000	-7400000000	9890000000
Lending Rate	330	20.32	4.73	1175
World Trade Share	372	0.0005	0	0.0063
Composite Trade Share	372	0.038	0	0.44
**ECOWAS**				
Trade Openness	180	72.43393	20.72	311.35
Economic Growth	179	3.931439	-20.59	20.71
Exchange Rate	180	978.4822	0.92	9100
Foreign Reserve	114	161000000	0.02	617000000
Foreign Direct Investment	180	842000000	-73800000	8840000000
Lending Rate	153	9.83	4.73	29.75
World Trade Share	180	0.0004	0	0.005
Composite Trade Share	180	0.0218994	0	0.23
**SADC**				
Trade Openness	190	89.28451	32.23894	225.0231
Economic Growth	192	4.585651	-17.66895	19.67532
Exchange Rate	192	357.2786	1	3176.539
Foreign Reserve	188	5380000000	40800000	45500000000
Foreign Direct Investment	192	841000000	-7400000000	9890000000
Lending Rate	177	29.40218	6.875	1175
World Trade Share	192	0.0006956	0	0.0063889
Composite Trade Share	192	0.0512485	0	0.4410274

Table 2 shows that trade openness has an average value of 81.08 for the combined dataset (ECOWAS and SADC), while for the other measures of trade openness, the world trade share and composite trade share both had an average of 0.0005 and 0.038. The combined dataset also reported a minimum value of 20.72 and a maximum value of 311.35 for trade to GDP ratio, while world trade share and composite trade share reported minimum values of 0 and maximum values of 0.063 and 0.44 respectively. Furthermore, the independent variables: growth rate, exchange rate, foreign reserve, foreign direct investment and lending rate, had average values of 4.27 percent, 657.86 LCU to USD, 3,410,000,000 USD, 841,000,000 USD and 20.32 percent. The minimum and maximum values of the variables, as shown in Table 2 reveal that economic growth rate also grew to 20.71 percent and was at the lowest point at -20.59 percent. Exchange rate also rose as high as 9100 LCU to USD and as low as 0.92 LCU to USD. Further, foreign reserve had its maximum value at 45,500,000,000 USD, which signifies how much foreign reserve grew, and its minimum value was as low as 0.02 USD. Foreign direct investment increased as much as 9,890,000,000and was at its base at -7,400,000,000 USD and finally lending rate had its peak at 1175 percent and its lowest value at 4.73 percent.

For the descriptive statistics of ECOWAS dataset, Table 2 indicated that trade to GDP ratio reported an average value of 72.4 percent; a minimum value of 20.7 percent and a maximum value of 311.4 percent, while for the other measures of trade openness, the world trade share and composite trade share both had an average of 0.0004 and 0.021; a minimum of 0 and 0; and a maximum of 0.005 and 0.238. The data for the independent variables, economic growth rate, exchange rate, foreign reserve, foreign direct investment and lending rate, had mean values of 3.9 percent, 978.5 LCU to USD, 161,000,000 USD, 842,000,000 USD, and 9.8 percent, respectively. The maximum and minimum values of the variables, as shown in Table 2 reveals that economic growth rate grew as high as 20.7 percent and was at the lowest at -20.7. Exchange rate was at its highest point at 9100 LCU to USD, and was at its lowest as -0.9 LCU to USD. Further, foreign reserve had its maximum value at 617,000,000 USD, which signifies how much foreign reserve grew, and it was as low as 0 USD. Foreign direct investment increased as much as 8,840,000,000 USD and was at its base at -73,800,000; while lending rate had its peak at 29.8 percent and its lowest value at 4.7 percent.

For SADC, Table 2 showed that trade to GDP ratio reported an average value of 89.3 percent; a minimum value of 32.2 percent and a maximum value of 225.0 percent, while for the other measures of trade openness, the world trade share and composite trade share both had an average of 0.0006 and 0.051; a minimum of 0 and 0; and a maximum of 0.006 and 0.441. The data for the independent variables, economic growth rate, exchange rate, foreign reserve, foreign direct investment and lending rate, had mean values of 3.9 percent, 978.5 LCU to USD, 161,000,000 USD, 842,000,000 USD, and 9.8 percent, respectively. The maximum and minimum values of the variables, as shown in Table 2 reveals that economic growth rate grew as high as 19.7 percent and was at the lowest at -17.7. Exchange rate was at its highest point at 3176 LCU to USD, and was at its lowest as 1.0 LCU to USD. Further, foreign reserve had its maximum value at 45,500,000,000 USD, which signifies how much foreign reserve grew, and it was as low as 40,800,000 USD. Foreign direct investment increased as much as 9,890,000,000 USD and was at its base at -7,400,000,000; while lending rate had its peak at 1175.0 percent and its lowest value at 6.9 percent.

### 4.2 Correlation analysis

As a primary estimation process, a correlation test was carried out, which generated the correlation matrix. The correlation matrix in Table 3 shows how the variables are correlated. Nevertheless, the correlation analysis coefficient for the regressors reveal that there is no issue of multicollinearity all ECOWAS and SADC countries together and individually as communities.

**Table 3:** Correlation matrix.

Variable	Economic Growth	Exchange Rate	Foreign Reserve	Foreign Direct Investment	Lending Rate
**ALL**					
Economic Growth	1.00				
Exchange Rate	-0.00	1.00			
Foreign Reserve	0.26	-0.19	1.00		
Foreign Direct Investment	0.06	-0.09	-0.14	1.00	
Lending Rate	0.15	-0.15	0.42	0.16	1.00
**ECOWAS**					
Economic Growth	1.00				
Exchange Rate	0.05	1.00			
Foreign Reserve	0.33	-0.09	1.00		
Foreign Direct Investment	-0.00	-0.21	-0.45	1.00	
Lending Rate	0.15	-0.37	0.75	0.14	1.00
**SADC**					
Economic Growth	1.00				
Exchange Rate	-0.00	1.00			
Foreign Reserve	0.26	-0.19	1.00		
Foreign Direct Investment	0.06	-0.09	-0.14	1.00	
Lending Rate	0.15	-0.15	0.42	0.16	1.00

### 4.3 Econometric results

This section presents the results based on the analysis carried out using the following econometric methods, Pooled Ordinary Least Squares, fixed effect model, random effects and some appropriateness checks. The analysis is carried out to examine the effects of the variables (economic growth rate, exchange rate, foreign reserve, foreign direct investment and lending rate, including the REC) on trade openness for both ECOWAS and SADC combined as well as individually. The rule of thumb for deciding which estimation is to be interpreted among the fixed and random effect results is determined by the Hausman test (Ejemeyovwi et al., 2019). The study further tests for the existence of endogeneity in the model, using the Durbin score and Wu-Hausman endogeneity test and carries out the Breusch-Pagan Lagrange Multiplier test for random effects to help decide between the pooled OLS result and the random effects results.

The preconditional appropriateness checks utilised by this study are the endogeneity tests, Breusch-Pagan Lagrange Multiplier (BPLM) random effect test and the Hausman test. Taking a look at the Durbin Score p-value and the Wu-Hausman p-value in the combined dataset (ECOWAS and SADC) shown in Table 4, the values are 0.53 and 0.54 respectively, which imply the absence of endogeneity in the empirical model due to the p-value being greater than 0.05 (statistically insignificant). An observation of the BPLM test indicated the non-suitability and hence, rejection of the Pooled OLS result as well as acceptance of the random effect model result. The BPLM findings lay the foundation for the Hausman test result to help decide the most appropriate technique between fixed and random effect models. The Hausman test result confirmed that the appropriate result for interpretation is the fixed effect model result, given the Hausman test p-value of 0.00.

**Table 4:** Estimation results (combined dataset).

Dependent Variable: Trade-GDP ratio	POLS	FEM	Robust FEM	REM
Economic Growth	∗∗∗0.05	∗0.03	0.03	0.02
	(0.08)	(0.03)	(0.12)	(0.12)
Exchange Rate	-0.083	0.14	0.14	-0.20
	(0.00)	(0.13)	(0.21)	(0.33)
Foreign Reserve	0.012	-0.05	-0.05	0.00
	(0.02)	(0.04)	(0.34)	(0.44)
Foreign Direct Investment	-0.01	0.06	0.06	0.04
	(0.44)	(0.00)	(0.14)	(0.00)
Lending Rate	0.04	-0.03	0.03	0.009
	(0.47)	(0.57)	(0.64)	(0.86)
RECdum	0.04			0.0008
	(0.19)			(0.99)
Constant	2.05	1.05	1.47	1.47
	(0.00)	(0.00)	(0.04)	(0.00)
R-square	0.29	0.49	0.23	0.24
F-statistic p-value	0.00	0.00	0.01	
Wald p-value				0.03
Durbin Score p-value	0.53			
Wu-Hausman p-value	0.55			
BPLM REM p-value	0.00			
BPLM REM decision	reject			accept
Hausman Test P-value		0.00		
Hausman decision		accept		reject

Note: p-values are in round brackets (), and ∗, ∗∗ and ∗∗∗ connotes 1%, 5%nd 10% levels of significance; POLS: Pooled OLS; FEM: Fixed Effects Model; REM: Random Effects Model; BPLM: Breusch-Pagan Lagrange Multiplier Test.

For the relationship between economic growth and trade openness, the robust fixed effect results in Table 4 reported an estimated 0.03 units, precisely. This coefficient implies that an increase of 1 percent in economic growth will lead to a less than proportionate positive effect of 0.03 percent. However, the relationship is statistically insignificant at 5 and 10 percent levels of significance. Similar results can be found for all the other variations, but the fixed effect result. The fixed effect reported a significant value, but it is not reliable because the robust version ensures that there is heteroscedasticity in the findings; hence, it can be concluded that for the combined regional economic communities of ECOWAS and SADC, economic growth does not transcend significantly into trade openness.

Concerning the specific results for ECOWAS and SADC regional economic communities shown in Table 5, the preconditional appropriateness checks such as the Durbin Score p-value and the Wu-Hausman p-value showed values of 0.93 and 0.94 for ECOWAS and 0.37 and 0.39 for SADC respectively.

**Table 5:** Estimation results (ECOWAS and SADC).

Estimators	POLS		FEM		REM		Robust REM	
	ECOWAS	SADC	ECOWAS	SADC	ECOWAS	SADC	ECOWAS	SADC
Economic Growth	∗∗∗0.05	0.05	0.04	∗∗0.033	0.03	0.02	0.03	0.02
	(0.09)	(0.19)	(0.18)	(0.05)	(0.22)	(0.13)	(0.23)	(0.24)
Exchange Rate	-0.07	-0.11	0.31	0.03	-0.08	-0.02	-0.08	-0.02
	(0.00)	(0.00)	(0.08)	(0.46)	(0.00)	(0.51)	(0.05)	(0.64)
Foreign Reserve	0.04	-0.01	-0.21	0.035	0.04	0.04	0.04	0.04
	(0.00)	(0.61)	(0.00)	(0.21)	(0.00)	(0.09)	(0.05)	(0.13)
Foreign Direct Investment	0.001	-0.004	0.05	0.06	-0.008	0.06	-0.008	0.06
	(0.95)	(0.86)	(0.22)	(0.00)	(0.78)	(0.00)	(0.91)	(1.87)
Lending Rate	-0.60	0.19	-0.86	0.08	-0.59	0.06	-0.5	0.06
	(0.00)	(0.02)	(0.08)	(0.07)	(0.00)	(0.13)	(0.00)	(0.35)
Constant	2.42	2.02	2.57	0.84∗	2.51	0.95	2.5	0.95
	(0.00)	(0.00)	(0.00)	(0.00)	(0.00)	(0.00)	(3.09)	(0.01)
R-square	0.72	0.25	0.23	0.18	0.20	0.17	0.17	0.15
F-statistic p-value	0.00	0.00	0.00	0.00				
Wald p-value					0.00	0.00	0.00	0.00
Durbin Score p-value	0.93	0.37						
Wu-Hausman p-value	0.94	0.39						
BPLM REM p-value	0.01	0.00			0.01	0.00		
BPLM REM decision	reject	reject			accept	accept		
Hausman Test p-value			0.25	0.50	0.25	0.50		
Hausman decision			reject	reject	accept	accept		

Note: p-values are in round brackets (), and ∗, ∗∗ and ∗∗∗ connotes 1%, 5%nd 10% levels of significance; POLS: Pooled OLS; FEM: Fixed Effects Model; REM: Random Effects Model.

The statistically insignificant values imply the absence of endogeneity in the individual empirical model. An observation of the BPLM test also indicates the non-appropriateness of the Pooled OLS result as well as acceptance of the random effect model result. This also lays the foundation for the Hausman test result to help decide the most appropriate technique between fixed and random effect techniques. The Hausman test result confirmed that the proper output for interpretation is the random effect model results for ECOWAS and SADC. The p-values of the Hausman tests were 0.25 and 0.50, respectively.

Having met all the preconditions and selection-appropriateness checks, the robust random effect technique is interpreted. Upon examining the existing relationship between economic growth and trade openness, specifically in ECOWAS and SADC, Table 4 reported coefficients of 0.03 percent and 0.02 percent respectively units. The coefficients imply that an increase of 1 percent in economic growth will lead to a less than proportionate positive effect of 0.03 percent in ECOWAS and 0.02 in SADC. However, the nexus is also statistically insignificant at 5 and 10 percent levels of significance. Similar results can be found for all the other variations, but the fixed effect result of SADC. The fixed effect reported a significant value, but it is not reliable because the Hausman test suggests that the random effect model is the appropriate result. Thus, on an individual basis, it can also be concluded that in the regional economic communities of ECOWAS and SADC, economic growth does not transmit significantly into trade openness.

In relation to both the combined RECs and the individual RECs findings, it was observed that between economic growth and trade openness, a statistically insignificant relationship was estimated at 5 percent level of significance. This finding goes against economic expectations which assumed that economic growth should transmit significantly to drive and encourage trade openness. This could be as a result of the fact that most ECOWAS and SADC countries that are involved in trade such as Nigeria, Mali, and Niger, hardly have developed industries to create substantial value from attained economic growth to export and further reap the fruits of trade openness such as the increase in the demand for a domestic country's currency (Ejemeyovwi et al., 2018). Zeren and Ari (2013) opined that an increase in countries' growth should increase openness in return. Therefore, high economic performance is considered among the factors influencing trade openness accordingly.

Despite the positive influence of growth rate in both RECs, they experience insignificance because they are not immune to the African continent's challenges. The International Labour Organisation (ILO) (2016), recognises high unemployment and underemployment as the challenges of growth in Africa. The rate of unemployment and underemployment contribute greatly to the increase or decrease in production and if the rates are too high, it will cause a reduction in the amount of total production. If the amount of total production is affected, it will affect general foreign trade and openness to trade. The exchange rate for ECOWAS was found to be positive and significant, while SADC was negative and insignificant. The insignificance may be attributed to policy shocks and also other macroeconomic shocks, both internal and external (Senadza and Diaba, 2017). Countries in this region like Nigeria, according to Olokoyo, Osabuohien and Salami (2009) have a low productive base, which affects the effectiveness and positive gains of trade in ECOWAS despite trade openness.

### 4.4 Robustness checks

To further confirm the findings of the study, robustness checks were employed following the pattern of Ho and Iyke (2019). The study utilises other composite measures of trade openness, given that trade to GDP ratio was critiqued to only capture the volume of trade within a country and not capture the interconnectedness of and interactions of the countries involved in trade (Iyke, 2017). Hence, this study employs the world trade share and the composite trade share, which accounts for the two dimensions of trade openness and trade intensity. Notably, the word trade share captures a country's relative contribution to the total world trade, while the composite trade share captures a country's trade share to overall economic activity, both developed by Squalli and Wilson (2011).

Table 6 presents the robustness test results for the combined ECOWAS and SADC regional economic blocks.

**Table 6:** Estimation results (ECOWAS and SADC).

Estimators	POLS	POLS	FEM	REM	FEM	REM
Dependent Variable	WTS	CTS	WTS	WTS	CTS	CTS
Economic Growth	∗52.16	∗46.31	0.000005	0.00005	0.0007	0.0007
	(0.00)	(0.01)	(0.16)	(0.16)	(0.06)	(0.05)
Exchange Rate	1.13	-4.53	-0.0000001	-.00000002	-0.00006	-0.00002
	(0.88)	(0.55)	(0.79)	(0.64)	(0.72)	(0.51)
Foreign Reserve	-3.62	-6.12	-0.0000027	-.00000014	-0.00014	-0.00009
	(0.28)	(0.07)	(0.06)	(0.81)	(0.03)	(0.13)
Foreign Direct Investment	-25.37 (0.004)	-34.30 (0.00)	-0.000023 (0.67)	.00000025 (0.01)	0.00002 (0.03)	0.000002 (0.19)
Lending Rate	90.56	136.21	0.00006	.00000003	-0.00001	0.000015
	(0.01)	(0.00)	(0.67)	(0.0003)	(0.47)	(0.46)
REC Dummy	-43.32 (0.02)	-51.55 (0.00)		.000611 (0.23)s		0.010 (0.64)
Constant	287.13	353.60	-.0004	.0002	0.031	0.95
	(0.00)	(0.00)	(0.00)	(0.27)	(0.00)	(0.00)
R-square	0.26	0.16	0.11	0.10	0.20	0.18
F-statistic p-value	0.00	0.00	0.00		0.00	
Wald p-value				0.09		0.00
Hausman Test p-value			0.89	0.89	0.78	0.78
Hausman decision			reject	accept	reject	accept

Note: p-values are in round brackets (), and ∗, ∗∗ and ∗∗∗ connotes 1%, 5%nd 10% levels of significance; POLS: Pooled OLS; FEM: Fixed Effects Model; REM: Random Effects Model; WTS: World Trade Share CTS: Composite Trade Share.

The table shows that while capturing the interconnectedness among the countries in world trade, economic growth was observed to drive trade openness, using the Pooled OLS estimator, while with the fixed and random effects results, trade openness was also positive but statistically insignificant at 5 percent level of significance. However, based on the panel nature of the study, this study is inclined to accept the decisions from the random and fixed effect results, over the pooled OLS result. Furthermore, among the fixed effect and random effect model, the Hausman test recommends the acceptance of the random effect estimators. In sum, the interconnectedness trade openness nexus with economic growth confirmed the findings of a positive but statistically insignificant result.

## 5 Conclusion

The study was motivated by the argument on the essence of the economic growth of trade openness in Africa, looking at the countries that belong to regional economic communities (RECs). The selected RECs for the study were ECOWAS and SADC countries from the period 2006 to 2017. For robustness purpose, the study utilised three measures of trade openness, namely trade to GDP ratio, world trade share and composite trade share. The study finds that economic growth does not significantly affect trade openness in the combined RECs as well as ECOWAS and SADC. The study is considered vital because of Africa's challenges with a low rate of intra-regional trade amongst its countries. This study argues that an improvement in economic conditions of nations will bring about improvement that will make available the vital structure that will enable the countries to embrace trading more with each other and motivate them to be more open to trade as well as contribute significantly, because, in this time and age of globalisation, autarky is outdated and improper.

Based on the findings noted in the previous section, it is recommended that efforts should be made to ensure that economic growth transmits significantly into trade openness. The African Continental Free Trade Area (AfCFTA) is aimed at fostering trade activities in the African continent as a whole. However, governments in ECOWAS and SADC need to embrace productive base increase and other solutions to ensure that countries have what to offer, thus, allowing significant economic growth's transmission to trade openness and globalisation. As a suggestion for further studies, the other impediments to the transmission of economic growth to change openness should be examined, such as the political institutions because the nations' macroeconomic condition should be sufficiently developed to enable trade openness in ECOWAS and SADC significantly.

## Declarations

### Author contribution statement

Betsy O. Moroyin: Performed the experiments; Wrote the paper.

Evans S. Osabuohien: Conceived and designed the experiments; Wrote the paper.

Jeremiah O. Ejemeyovwi: Analyzed and interpreted the data; Contributed reagents, materials, analysis tools or data; Wrote the paper.

### Funding statement

This research did not receive any specific grant from funding agencies in the public, commercial, or not-for-profit sectors.

### Data availability statement

Data included in article/supplementary material/referenced in article.

### Declaration of interests statement

The authors declare no conflict of interest.

### Additional information

No additional information is available for this paper.

## Figures and Tables

**Table A1:** List of Countries in ECOWAS and SADC RECs

ECOWAS Countries	SADC Countries
Benin	Angola
Burkina Faso	Botswana
Cote D'Ivoire	Comoros
Cabo Verde	DR Congo
The Gambia	Eswatini
Ghana	Lesotho
Guinea	Madagascar
Guinea Bissau	Malawi
Liberia	Mauritius
Mali	Mozambique
Niger	Namibia
Nigeria	Seychelles
Senegal	South Africa
Sierra Leone	Tanzania
Togo	Zambia
	Zimbabwe
